# Critical Role of TLR4 in Human Metapneumovirus Mediated Innate Immune Responses and Disease Pathogenesis

**DOI:** 10.1371/journal.pone.0078849

**Published:** 2013-10-29

**Authors:** Thangam Sudha Velayutham, Deepthi Kolli, Teodora Ivanciuc, Roberto P. Garofalo, Antonella Casola

**Affiliations:** 1 Department of Pediatrics, University of Texas Medical Branch, Galveston, Texas, United States of America; 2 Microbiology and Immunology, University of Texas Medical Branch, Galveston, Texas, United States of America; 3 Sealy Center for Vaccine Development, University of Texas Medical Branch, Galveston, Texas, United States of America; University of North Dakota, United States of America

## Abstract

Human metapneumovirus (hMPV) is one of the main causes of acute respiratory tract infections in children, elderly and immunocompromised patients. The mammalian Toll-like receptors (TLR) were identified as critical regulators of innate immunity to a variety of microbes, including viruses. We have recently shown that hMPV-induced cytokine, chemokine and type I interferon secretion in dendritic cells occurs via TLR4, however, its role in hMPV-induced disease is unknown. In this study, wild-type(WT) and TLR4-deficient mice (TLR4^−/−^) were infected with hMPV and examined for clinical disease parameters, such as body weight loss and airway obstruction, viral clearance, lung inflammation, dendritic cell maturation, T-cell proliferation and antibody production. Our results demonstrate that absence of TLR4 in hMPV-infected mice significantly reduced the inflammatory response as well as disease severity, shown by reduced body weight loss and airway obstruction and hyperresponsiveness (AHR), compared to WT mice. Levels of cytokines and chemokines were also significantly lower in the TLR4^−/−^ mice. Accordingly, recruitment of inflammatory cells in the BAL, lungs, as well as in lymph nodes, was significantly reduced in the TLR4^−/−^ mice, however, viral replication and clearance, as well as T-cell proliferation and neutralizing antibody production, were not affected. Our findings indicate that TLR4 is important for the activation of the innate immune response to hMPV, however it does play a role in disease pathogenesis, as lack of TLR4 expression is associated with reduced clinical manifestations of hMPV disease, without affecting viral protection.

## Introduction

Human metapneumovirus (hMPV), a member of the *Paramyxoviridae* family, was first identified in 2001 in Netherlands and since then it has been shown to be responsible for a significant proportion of all respiratory infections in children, elderly and immunocompromised patients [1]–[7]. In children it accounts for 5–15% of all the hospitalizations for lower respiratory tract infections and, in elderly patients, hMPV outbreaks in long term care facilities accounts for a high mortality rate [6]–[11]. The clinical disease spectrum for hMPV varies from upper respiratory tract infection to bronchiolitis, pneumonia, flu-like symptoms as well as asthma exacerbation [12]. Specific therapies/vaccines and more efficient means of prevention for hMPV have not been developed yet, in part due to the lack of substantial knowledge of the mechanisms involved in hMPV recognition and activation of immune responses, contributing to disease pathogenesis and immunity.

Innate immune responses initiated by the host-pathogen interaction serve as the first line of defense against invading pathogens and tailors the adaptive immune responses. Activation of innate immunity depends on the recognition of pathogen-associated molecular patterns (PAMPs) that are specific for the pathogen, by the pattern recognition receptors (PRRs) present in the host [13], [14]. PRRs like Toll-like receptors (TLRs), present on the cell surface and endosomal compartments, have been shown to be activated by viral infections, leading to signal cascades that regulates the expression of proinflammatory and immune mediators [13]–[17]. TLR4 is activated by lipopolysaccharide (LPS) and other structurally unrelated microbial structures such as Chlamydial Hsp60, pneumolysin, DnaK from Francisella tularensis, Ebola virus glycoprotein, envelope proteins of mammary tumor virus, murine leukemia virus and respiratory syncytial virus (RSV) F protein, as well as endogenous mammalian “danger signals,” such as fibrinogen, fibronectin, low-molecular-weight oligosaccharide fragments of hyaluronan, surfactant protein A and HMGB1 [18]–[26]. RSV F protein-mediated signaling through the TLR4 axis was shown to be dependent on CD14 and the physical interaction with MD-2, a non-membrane-spanning protein that associates with the TLR4 ectodomain [27], [28]. HMPV infection is known to activate intracellular targets like the RIG-I/MDA5/-MAVS signaling pathway *in vitro* and *in vivo*, leading to the expression of proinflammatory and antiviral molecules [29]–[31]. The hMPV F protein was found to be a TLR2 and TLR4 agonist, able to elicit an inflammatory response in human monocytes [32]. We have recently shown that the hMPV glycoprotein G modulates secretion of proinflammatory cytokines, chemokines and type I IFN in dendritic cells (DCs) by affecting TLR4-dependent signaling [33], however, the functional role of TLR4 in hMPV-induced disease pathogenesis and immune responses *in vivo* is unknown.

In the current study, we show that hMPV infection of TLR4^−/−^ mice is associated with reduced body weight loss and airway obstruction, and lower levels of proinflammatory cytokines and chemokines, leading to lower recruitment of immune cells to broncholaveolar lavage (BAL), lung and mediastinal lymph nodes (MLN), and normal lymphocyte proliferation and antibody production, compared to wild type mice. Although there was a trend in increased viral replication, TLR4^−/−^ mice cleared the virus and responded to viral challenge similar to wild type mice, indicating a role of TLR4 in disease pathogenesis and innate immune responses, but not in the adaptive one.

## Materials and Methods

### Viral preparation and titer

The hMPV strain CAN97-83 was propagated in LLC-MK2 cells (ATCC, Manassas, VA) in MEM (without serum) containing 1.0 µg trypsin/ml and viral pools for mice inoculation were filtered using Millipore filters, as previously described [33]. Viral and cellular preparations were routinely tested for mycoplasma contamination by PCR and were used if they had <0.125 EU/ml endotoxin (by Limulus assay). Viral pool titers were determined by immunostaining, as previously described. For viral titration in the lung, lungs were processed as previously described [34] and titration performed by TCID_50_ [35].

### Mice infection protocol

All procedures involving mice in this study were carried out in strict accordance with the recommendations in the Guide for the Care and Use of Laboratory Animals of the National Institutes of Health. The protocol was approved by the Institutional Animal Care and Use Committee of the University of Texas Medical Branch at Galveston (Protocol: 0808049). Six to seven week-old female C57BL/10ScSnJ (wild type) and C57BL/10ScNJ (TLR4^−/−^) mice (Jackson, Bar Harbor, Maine) were inoculated intranasally (i.n.) with 10^7^ pfu of filtered hMPV (live or UV inactivated), in a total volume of 50 µl, under light anesthesia (combination of xylazine, 8 mg/kg, and Ketamine: 70 mg/kg) given intraperitoneally. Control mice were inoculated with the same volume of virus-free medium (referred to hereafter as mock infected). At the indicated times post-infection (p.i.), lungs were collected and processed for viral titration or flow cytometry [34]. BAL was performed on days 1, 2, 3, 5, and 7 p.i. to determine total and differential cell counts, as previously described [34].

### Bio-Plex and ELISA

Chemokines and cytokines were quantified in BAL fluid by Luminex-based Bio-Plex system (Bio-Rad Laboratories, Hercules, CA) according to the manufacturer's instructions. The lower limit of detection for all cytokines measured by this assay is 3 pg/ml. IFN-α/-β concentrations were determined by commercial enzyme-linked immunosorbent assays (ELISA), according to the manufacturer's instructions (PBL, Piscataway, NJ).

### Flow Cytometry of lung cells

For flow cytometry analysis, lungs and mediastinal lymph nodes (MLN) were collected at different time points after hMPV or mock infection and digested with collagenase. Cells were passed through nylon mesh to get a single cell suspension, and incubated with Fc block (anti-mouse CD16/CD32) to reduce nonspecific binding, for 30 min before addition of Abs (BD Pharmingen, San Diego, CA) against surface markers: anti-CD3ε, -CD4, -CD8β, (T cells); -CD19, B220 (B cells); DX5, NK1.1 (NK cells); -CD11c, -CD11b, MHCII, F4/80, Ly6G & Ly6C (Gr-1), CCR3 and -mPDCA1 (Miltenyi Biotec) (cDCs, pDCs, macrophages, neutrophils, eosinophils, and IKDCs). Relevant isotype control antibodies were used throughout. Samples were stained and analyzed with a FACS Canto flow cytometer (BD Pharmingen, San Diego, CA) and data was analyzed using the FlowJo Software (Tree Star).

### Isolation of lung DCs and analysis of activation markers

In a separate set of experiments, lungs were collected at different time points after hMPV infection to prepare single cell suspensions. Cells were labelled with CD11c microbeads (Miltenyi Biotec, Auburn, CA) for enrichment of mouse DCs using magnetic activated cell sorting (Auto MACS), and isolated cells were stained with anti-CD11c and anti-MHC-II, in combination with anti-CD40, -CD69, -CD80, -CD83 andCD86) (BD-Pharmingen, San Diego, CA). Samples were stained and analyzed with a FACS Canto flow cytometer and data was analyzed using the FlowJo Software (Tree Star).

### T cell Proliferation

For T cell proliferation assays, MACS sorted cDC from hMPV-infected and uninfected WT or TLR4^−/−^ mice (10^5^ cells/well) were cultured for 3 days with CD4^+^ T cells from OT-II mice (2×10^5^ cells/well) (previously tagged with CFSE) in 96-well round bottom microtiter plates. cDC were loaded with 10 µg/mL of OVA peptide (323–339, Genscript, Piscataway, NJ) 2 hrs prior to co-culture with T cells. After 3 days, cells were collected, washed and analyzed by FACS for T-cell proliferation, determined by increase in CFSE ^low^ cells.

### Clinical disease

Daily determination of body weight was used to monitor the progression of disease over a period of 12 days p.i. with hMPV.

### Microneutralization assay for measurement of serum anti-hMPV antibodies

Heat inactivated sera was tested for neutralizing antibodies to hMPV, as previously described [36]. The neutralizing antibody titers were defined as the reciprocal of the highest serum dilution at which ≥50% reduction in CPE was observed. The lowest detectable titer was 2.5 log_2_. Samples with non-detectable titers were assigned a value of 2 log_2_.

### Airway hyperresponsiveness (AHR)

AHR was assessed in unrestrained mice using whole-body barometric plethysmography (Buxco, Troy, NY) to record enhanced pause (Penh), as previously described [37]. Penh is a dimensionless value that represents a function of the ratio of peak expiratory flow to peak inspiratory flow and a function of the timing of expiration. Penh has previously been validated in animal models of AHR [38]–[41] and infection associated airway obstruction [42]. Respiratory activity was recorded for 4 min, to establish baseline Penh values. Mice were subsequently exposed to increasing doses of nebulized methacholine (3.125, 12.5, 25, and 50 mg/ml) for 2 min, and data were recorded for another 3 min.

### Respiratory mechanics

Invasive analysis of lung function was performed using the Flexivent system (Scireq, Montreal, Quebec, Canada) as previously described [43], [44]. Breifly, mice were anesthetized with xylazine and pentobarbital sodium (50 mg/kg of body weight), cannulated with an 18G needle (for the delivery of methacholine) and artificially ventilated at 150 breaths/min with a tidal volume of 0.3 ml and a positive end expiratory pressure of 3 cm H_2_O. Baseline pulmonary mechanics and responses to aerosolized methacholine (0 to 50 mg/ml) were then obtained by using the forced-oscillation technique.

### Statistical analysis

When two groups were compared, the values were analyzed using an unpaired, two-tailed Student's *t* test and when multiple groups were compared, ANOVA was used (GraphPad Instat Software, Inc., San Diego, CA). Separate independent samples t-tests were performed for each of the four experiments. Results are expressed as mean ± SEM unless otherwise stated.

## Results

### hMPV-induced clinical disease and viral replication in the lung

**Figure 1:**
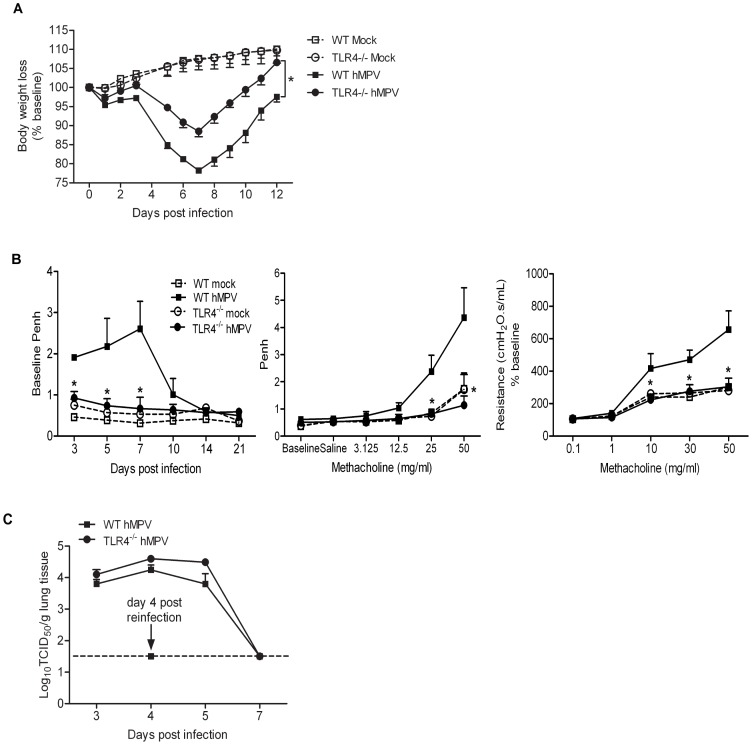
HMPV-induced clinical disease, lung function and viral replication in the absence of TLR4. TLR4^−/−^ and wild type (WT) mice were either infected with hMPV (live or UV inactivated) or mock infected. Change in body weight was measured over a period of 12 days. Body weight is expressed as percentage of baseline weight (**A**). Baseline Penh values were measured over a period of 21 days; post-methacholine challenge airway hyperresponsiveness and airway resistance were measured at day 14 p.i. (**B**). TLR4^−/−^ and WT mice were infected with hMPV and sacrificed at days 3, 4, 5 and 7 p.i. to determine viral titers by TCID_50_ assay. Mice were challenged with hMPV (10^7^ pfu) 6 weeks after primary infection and lung viral titers were determined on day 4 p.i. (peak of viral replication). The lower limit of detection of this assay is 1.5 log_10_/gram of tissue, represented by the dotted line (**C**). Data are expressed as mean ± SEM of four to six animals/group. Figure 1A and B represents one of three independent experiments and Figure 1C represents cumulative data from three independent experiments. *P<0.05 when comparing hMPV infected TLR4^−/−^ mice to WT mice.

WT and TLR4^−/−^ mice were infected with hMPV (live or UV inactivated) or mock infected and body weight loss was measured over the following 12 days. Both WT and TLR4^−/−^ infected mice showed an initial phase of body weight loss, followed by a second phase, starting around day 5 p.i. During both phases, WT mice exhibited significantly more body weight loss and a delayed recovery during the second phase, compared to TLR4^−/−^ mice (Fig. 1A). Infection with UV-inactivated hMPV failed to induce any body weight loss in the WT mice, behaving similar to the mock-infected group throughout the period of disease progression (Fig. S1A).

We have previously shown that hMPV infection in mice is associated with airway obstruction and hyperresponsiveness (AHR), using both unrestrained whole-body plethysmography and invasive analysis of lung function [43]. WT mice infected with hMPV showed consistently increased baseline obstruction compared to TLR4^−/−^ infected mice, starting at day 3 p.i., peaking around day 7 and returning to baseline by day 14 p.i. (Fig. 1B). They also exhibited a significant dose-dependent increase in airway hyperresponsiveness (AHR) in response to aerosolized methacholine, compared to TLR4^−/−^ mice, which exhibited values mostly similar to mock-infected animals (Fig. 1B). Increased airway resistance in response to inhaled methacholine in hMPV infected WT mice, compared to TLR4^−/−^ mice, was confirmed using invasive analysis of lung function on anesthetized mice using the Flexivent system (Scireq, Montreal, Quebec, Canada)(Fig. 1B).

To assess hMPV replication in the lung, TLR4^−/−^ and WT mice were sacrificed at day 3, 4, 5 and 7 p.i and titers determined by TCID_50_ assay. HMPV replicated efficiently in the lungs of WT and TLR4^−/−^ mice, with a peak of viral titer at day 4 p.i., and declined to undetectable levels by day 7 p.i. after infection (Fig. 1C). The amount of replicating virus detected in the lungs at various time points p.i., was not significantly different between the WT and TLR4^−/−^ mice and in both groups the virus was cleared efficiently from the lungs by day 7 p.i. To determine whether the absence of TLR4 affected protection from subsequent reinfection, we investigated viral replication in WT and TLR4^−/−^ mice challenged with hMPV six weeks after primary infection. Both mice challenged with hMPV had peak viral titers at the lower limit of detection (<1.5 log_10_ TCID50/g tissue), assessed at day 4 p.i., indicating that overall the absence of TLR4 does not have a significant impact on hMPV replication, clearance and ability to respond to a secondary infection.

### Inflammatory and immune mediator production

**Figure 2:**
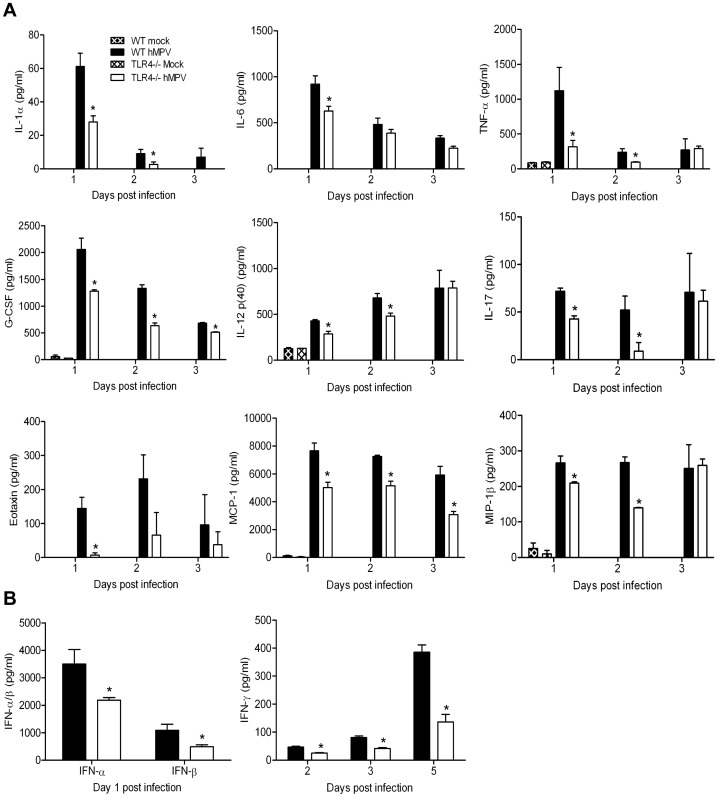
Expression of pro-inflammatory cytokine, chemokines and type I IFNs in response to hMPV infection of TLR4-deficient mice. TLR4^−/−^ and WT mice were either infected with hMPV or mock infected, and sacrificed at days 1, 2, 3, 5, 7 p.i. to collect BAL fluid. Levels of IL-1α, IL-6, TNF-α, G-CSF, IL-12 p(40), IL-17, Eotaxin, MCP-1, MIP-1β in BAL fluid were measured by Bio-Plex (**A**). Levels of IFN-α/β (left panel) were measured by ELISA on day 1 p.i., while IFN-γ (right panel) was measured by Bio-Plex at various days p.i. (**B**). Data are expressed as mean ± SEM of four to six animals/group and represents one of two independent experiments. *P<0.05 when comparing hMPV infected TLR4^−/−^ mice to infected WT mice.

To determine the role of TLR4 in the regulation of lung inflammatory response to hMPV infection, BAL fluid from both TLR4^−/−^ and WT mice infected with hMPV was collected at several days p.i. and assessed for levels of cytokines, chemokines and IFN-α/β production. As shown in Fig. 2A and 2B, TLR4^−/−^ mice had significantly lower levels of proinflammatory cytokines (IL-1α, IL-6, TNF-α), immunomodulatory cytokines (G-CSF, GM-CSF, IL-12p(40), IL-17) and chemokines (Eotaxin, MCP-1, MIP-1α)(Fig. 2A), as well as type I IFN (Fig. 2B, left panel) at day 1 p.i., compared to WT mice. Level of the Th1 regulatory cytokine IFN-γ was also significantly reduced in TLR4^−/−^ mice on various days p.i, in particular on day 5, which corresponds to peak levels of IFN-γ production in response to hMPV infection (Fig. 2B, right panel)[43]. There was no significant difference in the secretion of KC, RANTES, IL-4 or IL-5 between the WT and TLR4^−/−^ mice in response to hMPV infection (data not shown). UV-inactivated hMPV failed to induce a robust cytokine and chemokine response in BAL fluid 24 hrs p.i. compared to infection with live hMPV in WT mice (Fig. S1B).

### Immune cell trafficking

**Figure 3:**
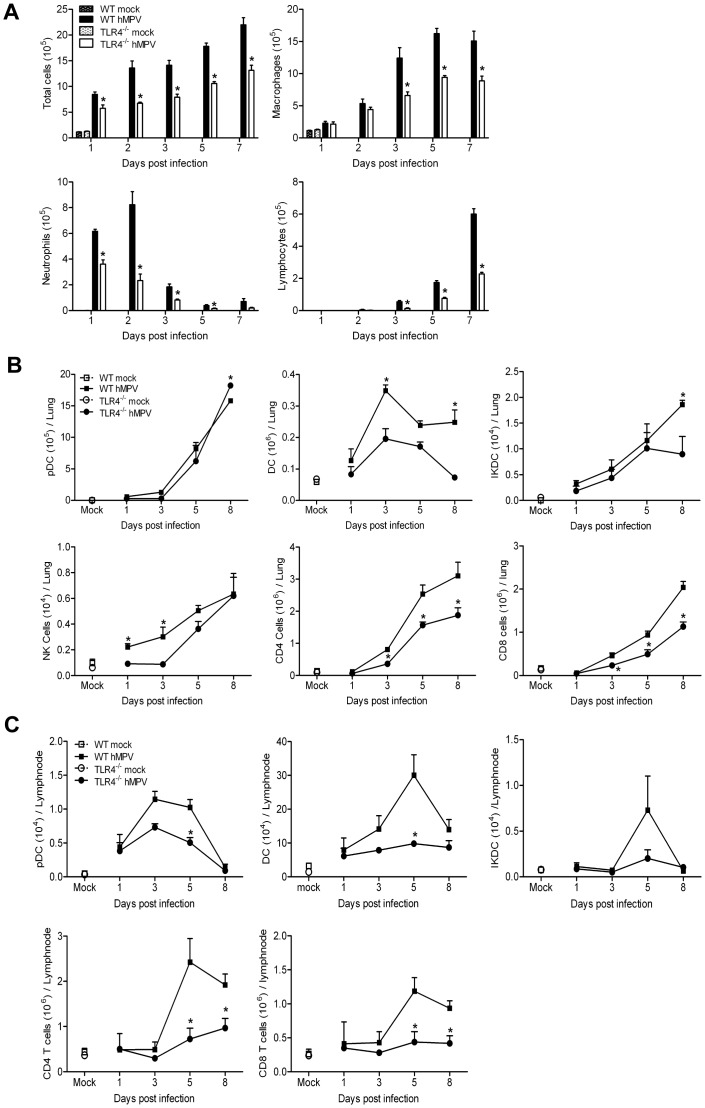
Recruitment of inflammatory cells in response to hMPV infection in BAL, lung and lymph nodes in TLR4^−/−^ mice. TLR4^−/−^ and WT mice were either infected with hMPV or mock-infected, and sacrificed at days 1, 2, 3, 5, 7/8 p.i. to collect BAL, lungs and mediastinal lymph nodes (MLN). Total and differential cell counts were determined in the BAL fluid (**A**). Cells isolated from lungs (**B**) and MLN (**C**) were stained with cell-type specific fluorochrome conjugated and processed by flow cytometry. Data are expressed as mean ± SEM of four mice/group and represents one of three independent experiments. *P<0.05 when comparing hMPV infected TLR4^−/−^ mice to infected WT mice.

TLR4^−/−^ and WT mice were infected with hMPV and BAL fluid, total lung tissue and mediastinal lymph nodes (MLN) were harvested up to day 7/8 p.i., which represents peak of lung inflammation [43]. In the absence of TLR4, the overall recruitment of inflammatory cells in BAL fluid was significantly reduced compared to that of the WT mice, in response to hMPV infection (Fig. 3A). BAL fluid from mock-inoculated mice consisted mainly of macrophages, whereas there was remarkable neutrophilia within the first 2 days of hMPV infection, which was significantly higher in WT mice compared to TLR4^−/−^ mice (Fig. 3A). The mononuclear cells, including monocytes/macrophages and lymphocytes, started to increase by day 3 p.i. and represented the majority of BAL cells until day 7 p.i., with absolute numbers being significantly higher in infected WT mice compared to the TLR4^−/−^ mice (Fig.3A). No eosinophils were observed in the BAL samples of any of the groups of mice analyzed. Flow cytometric analysis of cell populations from the lung and MLN shows that trafficking of plasmacytoid dendritic cells (pDC), conventional dendritic cells (cDC) and interferon producing killer dendritic cells (IKDC) to the lung and MLN after hMPV infection was also affected by the lack of TLR4 expression. While the number of lung pDCs was mostly similar between the TLR4^−/−^ and WT mice, with the exception of late time points of infection (Fig. 3B), the number of pDCs in MLN was significantly higher in WT mice, compare to the TLR4^−/−^ mice, during the acute phase of infection (day 5 p.i., Fig. 3C). The number of lung cDC was significantly higher in the WT mice, compared to the TLR4^−/−^ mice on days 3 and 8 p.i.(Fig. 3B), with a significant reduction of trafficking of cDC to MLN observed in TLR4^−/−^ mice mostly around the peak of recruitment, which occurred at day 5 p.i. (Fig. 3C). Recruitment of IKDCs mirrored the one observed for pDCs (Fig. 3B and C). NK cells were detected in the lungs of WT infected mice as early as day 1 p.i., increasing rapidly to peak at day 8 and declining thereafter, while NK cell recruitment in TLR4^−/−^ mice was significantly reduced and delayed, as it started only around day 5, to gradually peak by day 8 p.i., to reach levels similar to WT mice (Fig. 3B).

Both CD4^+^ and CD8^+^ T cells started to infiltrate the lungs of WT mice as early as day 3 p.i., increasing rapidly to peak at day 8 p.i. (Fig. 3B), and declining thereafter, with levels at day 15 p.i. still higher than in mock-infected groups (data not shown), while the kinetics of recruitment of these cells to the MLN showed a peak at day 5 p.i. (Fig. 3C), with a progressive decrease at later time points of infection. At various time points after infection, higher number of CD4+ and CD8+ T cells were recruited to the lungs and MLN of WT mice compared to TLR4^−/−^ mice, with significant differences observed from days 3 to 8 p.i. in the lungs and days 5 to 8 p.i. in the MLN compartment (Fig. 3B and 3C). Also the percentage of activated CD4 T cells (CD4^+^ CD69^+^) was significantly higher in the lungs of WT compared to the TLR4^−/−^ mice, as determined at day 8 p.i. (Fig. S2).

### Dendritic cell activation and T cell proliferation

**Figure 4:**
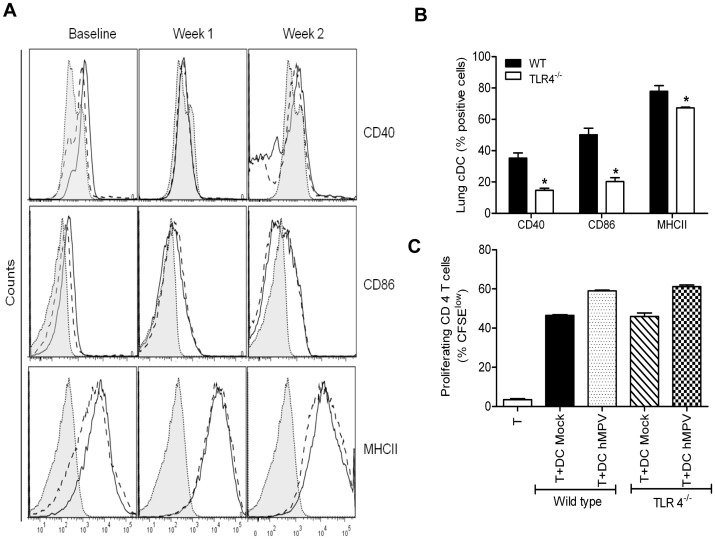
Lung dendritic cell characterization and lymphocyte proliferation in response to hMPV infection in TLR4^−/−^ mice. TLR4^−/−^ and WT mice were mock- or hMPV-infected, and sacrificed at week 1 and 2 p.i. to collect lungs. Single cell suspension was obtained and cDCs population enriched using CD11c-tagged magnetic beads isolation. CD40 and CD86 markers were analyzed in cells positive for CD11c and MHC-II by flow cytometry. Histograms showing the expression of costimulatory molecules (CD40, CD86 and MHCII) in cDC from WT mice (open histograms) and cDC from TLR4^−/−^ mice (dashed line open histograms), and isotype control (shaded histograms), are shown (**A**). Graph of baseline expression of costimulatory molecules in lung dendritic cells isolated from WT and TLR4^−/−^ mice (**B**). Dendritic cells (CD11c positive cells) were isolated from lungs of hMPV or mock-infected mice either WT or TLR4^−/−^ at day 7 p.i. and loaded with 10 µg/mL of OVA peptide for 2h prior to coculture with T cells. CD4^+^ T cells isolated from spleen of OT II mice were labeled with CFSE and cocultured with DCs at a ratio of 1∶2 (DC: T). T cell proliferation was measured by CFSE dilution (proliferating CD4^+^ cells have a lower CFSE intensity than non-proliferated control cells). Cultures without antigen served as controls. The bar graph shows the percentage of proliferating (CFSE ^low^) T cells among the total CD4^+^ T cell population. Data are expressed as mean ± SEM of four mice/group and represent one of two independent experiments (**C**).

To assess the impact of the lack of TLR4 on DC activation and function, lung CD11c positive cells were isolated from WT and TLR4^−/−^ mice using flow cytometry and assessed for the expression of costimulatory/activation markers, as well as their ability to induce T-cell proliferation *in vitro*. There was a significant difference in basal expression of CD40 (20% increased expression), CD86 (30% increased expression) and MHCII (11% increased expression) in DC isolated from WT mice compared to TLR4^−/−^ counterparts (Fig. 4A and B). Following hMPV infection, levels of these activation markers was similar between WT and TLR4^−/−^ mice at week 1 and 2 p.i. Expression levels of CD80, CD83 were similar at baseline and following infection in both groups of mice (data not shown). Overall, these data suggest that DCs were able to mature properly in response to hMPV infection in the absence of TLR4.

To investigate antigen presenting capacity, pulmonary DCs from WT and TLR4^−/−^ mice were isolated from lungs of hMPV- and mock-infected mice at week 1, 2 and 3 p.i., loaded with OVA peptide and co-cultured with CFSE labeled, purified splenic CD4^+^ T cells from OT-II mice. Incubation of lung DCs from mock and hMPV-infected mice with OT II CD4^+^ T cells resulted in a robust T cell proliferation, as indicated by the increase in percent of CFSE^low^ cells (Fig. 4C), with higher proliferation observed in cultures of DCs isolated from hMPV infected mice. There was no significant difference between the WT and TLR 4^−/−^ mice in their capability to induce T cell proliferation. Similar results were obtained at week 2 and 3 p.i. (data not shown).

### Serum neutralizing antibody production

**Figure 5:**
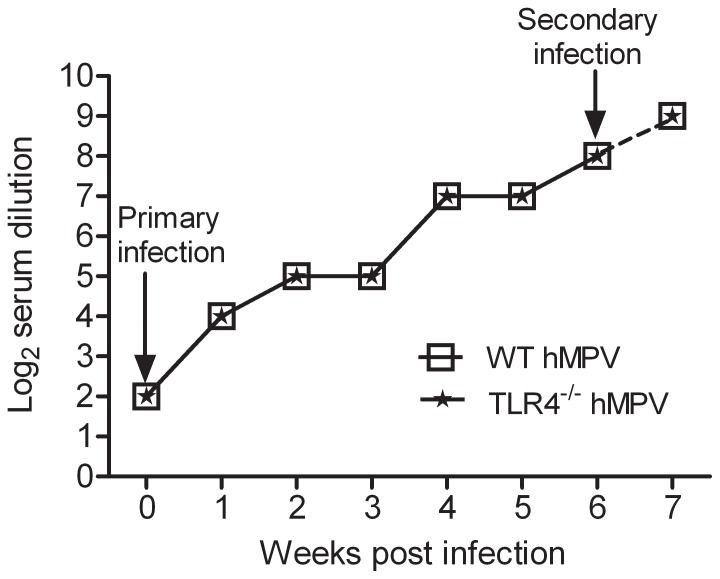
Induction of hMPV-specific neutralizing antibodies in the absence of TLR4. TLR4^−/−^ and WT mice were infected with hMPV and sacrificed weekly to determine antibody titers by a plaque reduction neutralization assay. The lower detection limit for this assay is 2 log_2_ serum dilution. At 6 weeks p.i. mice were challenged with hMPV and neutralizing antibody titers were determined 1 week later. Data are expressed as mean of four mice/group and represents one of two independent experiments.

Neutralizing antibody was first detected at week 1 p.i. The titer progressively increased during the following weeks, peaking between 4 and 6 weeks p.i., with the titer being similar in WT and TLR4^−/−^ infected mice (Fig. 5). To determine whether animals responded similarly to reinfection, mice were challenged with hMPV six weeks after primary infection, and serum was collected one week p.i. to determine antibody titers. There was a twofold increase in neutralizing antibody titers in both WT and TLR4^−/−^ mice challenged with hMPV, compared to the antibody levels present at the time of the challenge (Fig. 5). These results show that the absence of TLR4 does not affect the production of protective neutralizing antibodies in response to primary and secondary hMPV infection.

## Discussion

Respiratory viruses are the most frequent cause of the acute respiratory tract infections in children and the outcome of these respiratory viral infections depends on a combination of the host immune responses and virus-induced cellular damage. PRR-mediated immune responses have been implicated in both host protection as well as in immunopathology. Among the PRRs, TLRs are integral components of lung immune function during health and disease processes. In recent investigations, we have shown that TLR4 plays an important role in hMPV-induced secretion of proinflammatory cytokines and chemokines, as well as type I IFN in moDCs [33]. In the present study, we have examined the role of TLR 4 in hMPV-mediated disease, as well as immune responses, and found that TLR4 plays an important role in the regulation of hMPV-induced inflammatory responses and disease pathogenesis *in vivo*.

Our results show that mice lacking TLR4, infected with hMPV, showed less clinical disease, demonstrated by significantly reduced body weight loss and less airway obstruction and AHR, compared to WT mice. Significantly lower levels of proinflammatory cytokines (IL-1β, IL-6 and TNF-α), immunomodulatory cytokines (GM-CSF, IL-12 p40, IL-17) and chemokines (MCP-1, MIP-1α) were detected in the TLR4^−/−^ infected mice, compared to the WT. Accordingly, inflammatory cell recruitment in the BAL, lungs, as well as in lymph nodes, was also significantly reduced. Also, infection with UV-inactivated hMPV failed to induce significant body weight loss and secretion of cytokines/chemokines in BAL fluid in the WT mice, demonstrating that they both require active viral replication. However, there was no significant effect on viral replication and clearance from the lungs, ability to respond to viral challenge, DC activation and maturation and production of neutralizing antibodies. These results indicate that TLR4 is important for activation of the innate immune response to hMPV infection, however, it also contributes to disease pathogenesis.

TLR4, the first discovered human homologue of the *Drosphila* Toll, has been shown to recognize and be activated by several viruses such as Ebola virus, Hanta virus, Herpes virus and RSV. Several investigators have examined the interaction between TLR4 and RSV, a pneumovirus belonging to the *Paramyxoviridae* family, similar to hMPV. Different from our findings, Kurt-Jones et al., using TLR4-deficient C57BL10/ScCr mice (TLR4 deleted mutant mice), reported a delayed clearance of the virus compared to WT mice [27]. Since the mice strain used had a defect in IL-12 signaling, the same group later repeated the study using TLR4-deficient mice with intact IL-12 signaling (C57BL10/ScNCr) and reported impaired NK cell and CD14+ cell pulmonary trafficking, diminished NK cell function, and reduced IL-12 induction, in addition to impaired viral clearance [45]. TLR4, together with the adaptor molecule MyD88, has also shown to be required for optimal protection against viral challenge in a mouse model of RSV infection [46]. While in this study we did not evaluated the role of MyD88 in hMPV primary and secondary infection, our results indicate that TLR4 is not necessary to protect against viral challenge. Recent investigations have also suggested a role of TLR4 in resolution of RSV-induced lung inflammation [47], and TLR4 mutations, leading to decreased TLR4 function, have been associated with increased severity of RSV bronchiolitis in infants [48]. All together, these data indicate a protective role of TLR4 in RSV infection.

We have previously reported that DCs isolated from TLR4 deficient mice show reduced inflammatory mediator secretion following hMPV infection [33]. Similarly, Murawski *et al.* [49] observed a blunted cytokine response in peritoneal macrophages obtained from TLR4-deficient mice upon RSV infection. In this study, we observed lower levels of proinflammatory cytokines and chemokines in the BAL of TLR4-deficient mice in response to hMPV infection, which was paralleled by a reduction in inflammatory and immune cell recruitment, but did not seem to have a major impact on DC activation and their ability to support T cell proliferation and antibody production, suggesting that TLR4 sensing/transduction does not influence these immune functions in response to hMPV infection. In fact, although we observed significantly lower baseline levels of the MHC II and costimulatory molecules, CD86 and CD40 on DCs in the absence of TLR4, the maturation and induction of these molecules was comparable to that of cells expressing TLR4 in response to hMPV infection. Similarly, there was no difference in the DC ability to induce T cell proliferation and no difference in the ability to mount a protective antibody response during primary and secondary hMPV infection, indicating that the lack of TLR4 primarily affects the initial proinflammatory response, while the protective immune response is preserved and sufficient for effective antiviral responses, suggesting the presence of multiple pathways leading to DC and T cell activation, in the absence of TLR4 activation during hMPV infection.

Although we cannot define the exact mechanisms responsible for hMPV-induced disease in the mouse model of infection, the blunted inflammatory response could be part of the reason for the observed reduced clinical disease, both in terms of body weight loss and airway function in TLR4^−/−^ mice. However, a reduced T cell lung infiltration could also have contributed to a better disease outcome, as we have previously shown that both CD4 and CD8 T cell play a role in pathogenesis of hMPV-mediated lung disease [43]. In conclusion, we found that TLR4, while play a significant pathogenic role in the initial phase of hMPV infection by contributing to the inflammatory response and clinical disease, it does not mediate adaptive responses important for viral clearance and protection against reinfection.

## Supporting Information

Figure S1 **Body weight loss and cytokines/chemokine secretion in response to UV-inactivated hMPV.** WT mice were either infected with hMPV or UV-inactivated hMPV or mock infected. Change in body weight was measured over a period of 12 days and is expressed as percentage of baseline weight (**A**). Levels of IL-1α, IL-6, TNF-α, G-CSF, IL-12 p(40), IL-17, Eotaxin, MCP-1, MIP-1β fluid were measured in BAL fluid on day 1 p.i. (**B**). Data are expressed as mean ± SEM of four animals/group. *P<0.05 when comparing mice infected with UV-inactivated versus live hMPV.
(TIF)

Figure S2 **Percentage of activated CD4 T cells recruited to the lungs at the peak of T cell infiltration.** TLR4^−/−^ and WT mice were infected with hMPV or mock-infected, and sacrificed at day 8 p.i. to collect lungs. Cells isolated from lungs were stained with anti-CD3, -CD4, and -CD69 antibodies and analyzed by flow cytometry to determine expression of CD69 which is represented as percentage of CD4^+^ T cells. Graph represents mean ± SEM of four mice/group. *P<0.05 when comparing CD4^+^ T cells isolated from hMPV infected TLR4^−/−^ mice versus WT mice.
(TIF)
