# Supplementary figures and images for: Critical Role of TLR4 in Human Metapneumovirus Mediated Innate Immune Responses and Disease Pathogenesis
Source: PLoS One. 2013 Oct 29;8(10):e78849. doi: 10.1371/journal.pone.0078849 (PMC3812158; doi:10.1371/journal.pone.0078849)

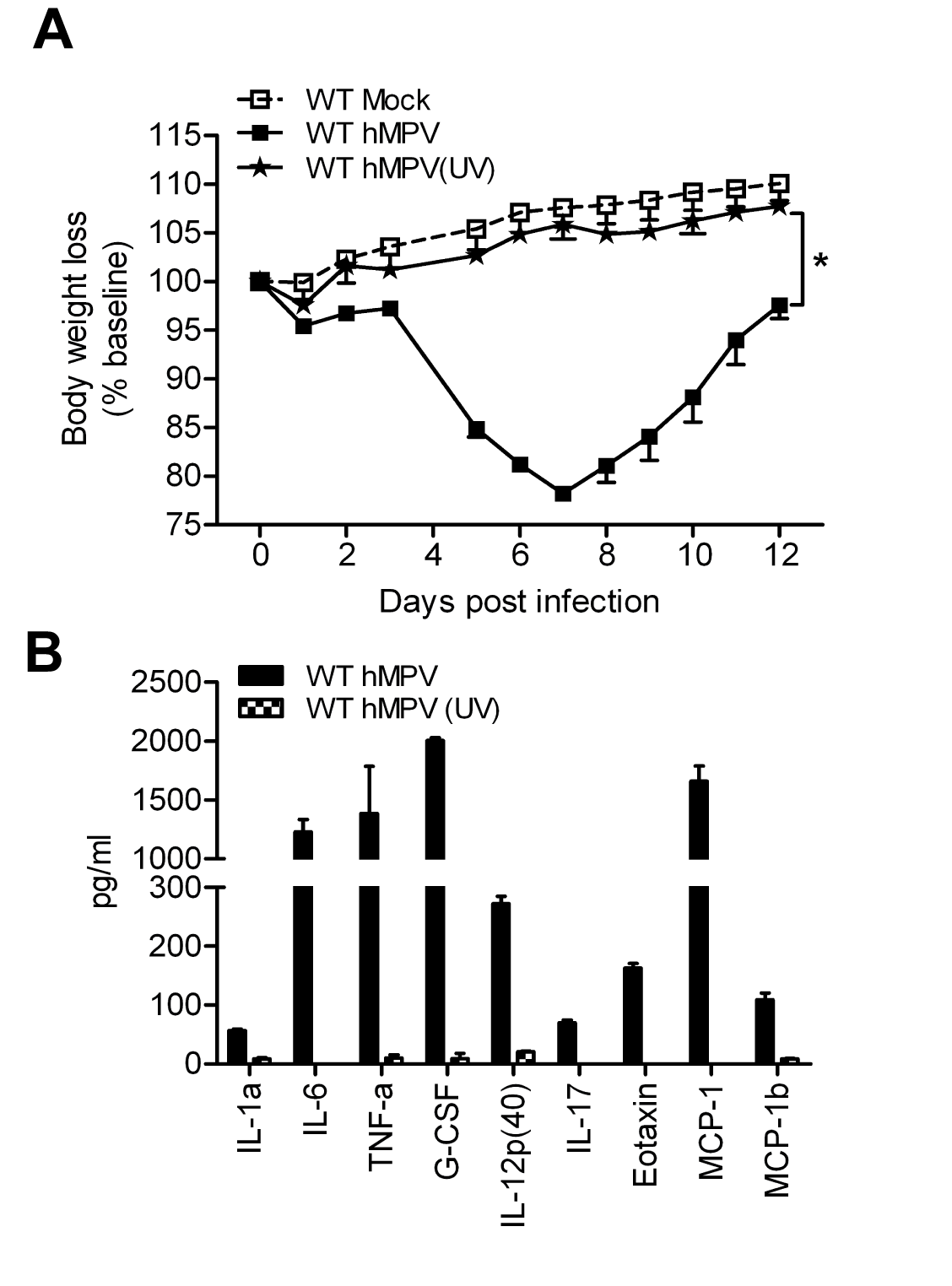

Supplement: Figure S1 — Body weight loss and cytokines/chemokine secretion in response to UV-inactivated hMPV. WT mice were either infected with hMPV or UV-inactivated hMPV or mock infected. Change in body weight was measured over a period of 12 days and is expressed as percentage of baseline weight (A). Levels of IL-1α, IL-6, TNF-α, G-CSF, IL-12 p(40), IL-17, Eotaxin, MCP-1, MIP-1β fluid were measured in BAL fluid on day 1 p.i. (B). Data are expressed as mean ± SEM of four animals/group. *P<0.05 when comparing mice infected with UV-inactivated versus live hMPV. (TIF) [file pone.0078849.s001.tif]

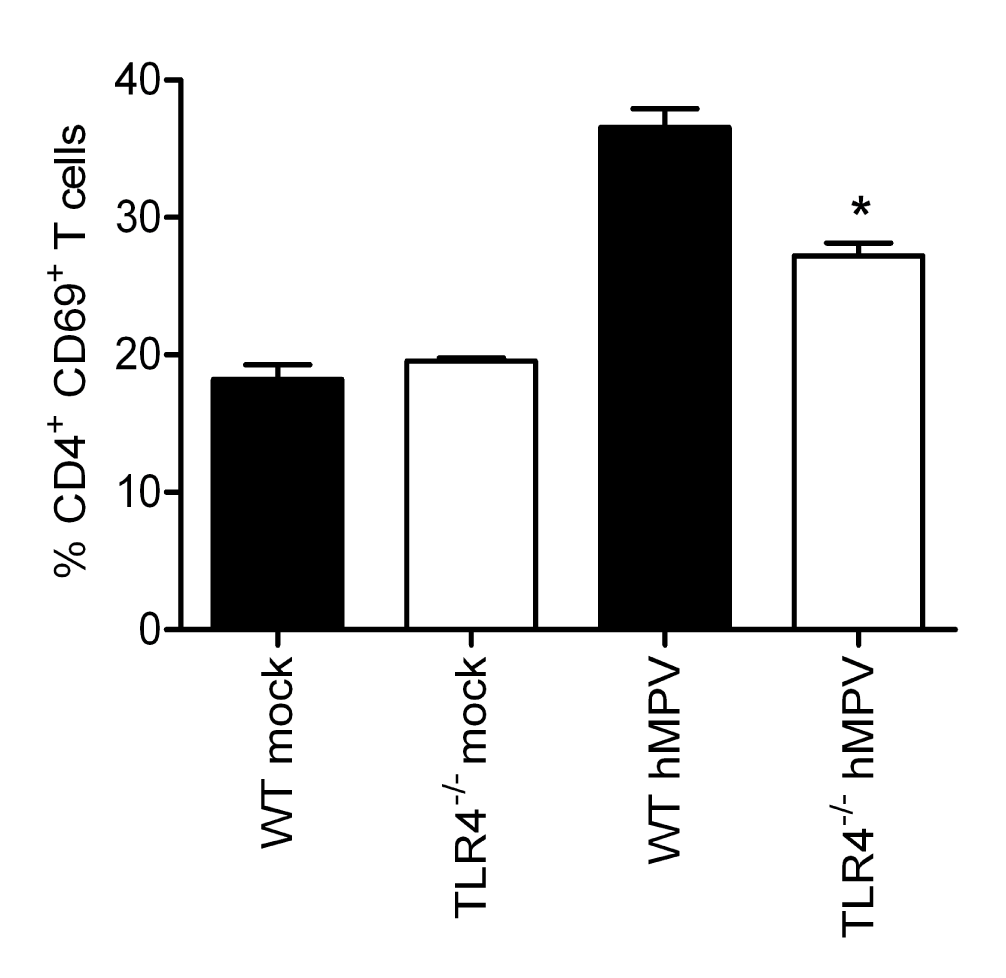

Supplement: Figure S2 — Percentage of activated CD4 T cells recruited to the lungs at the peak of T cell infiltration. TLR4−/− and WT mice were infected with hMPV or mock-infected, and sacrificed at day 8 p.i. to collect lungs. Cells isolated from lungs were stained with anti-CD3, -CD4, and -CD69 antibodies and analyzed by flow cytometry to determine expression of CD69 which is represented as percentage of CD4+ T cells. Graph represents mean ± SEM of four mice/group. *P<0.05 when comparing CD4+ T cells isolated from hMPV infected TLR4−/− mice versus WT mice. (TIF) [file pone.0078849.s002.tif]
